# The Role of an Assistant Nurse in Implementing the WHO Surgical Safety Checklist: Perception and Perspectives

**DOI:** 10.7759/cureus.38854

**Published:** 2023-05-10

**Authors:** Edin Ališić, Melissa Krupić, Jasmin Alić, Kemal Grbić, Nejra Mašić, Shariet Parvaneh, Ferid Krupić

**Affiliations:** 1 Department of Anesthesiology, Sahlgrenska University Hospital/Östra, Gothenburg, SWE; 2 Urology Clinic, Clinical Center University of Sarajevo, Sarajevo, BIH; 3 Clinic of Thoracic Surgery, Clinical Center University of Sarajevo, Sarajevo, BIH; 4 Neurology Clinic, Clinical Center University of Sarajevo, Sarajevo, BIH; 5 Department of Gynecology, Sahlgrenska University Hospital/Östra, Gothenburg, SWE; 6 Department of Anesthesiology/Orthopedics, Institute of Clinical Sciences, Sahlgrenska Academy at University of Gothenburg, Gothenburg, SWE

**Keywords:** world health organization, healthcare, assistant nurse, surgical safety checklist, surgery

## Abstract

Introduction

The World Health Organization (WHO) Surgical Safety Checklist is a tool developed by the WHO to promote safer surgical practices and reduce the incidence of surgical errors and complications. This study aims to describe the role of assistant nurses in the implementation of this checklist by surgical teams.

Materials and methods

This descriptive study utilized a questionnaire-based survey conducted between September 2018 and March 2019 among 196 healthcare professionals at two surgical units in a university hospital in Sweden. The questionnaire covered demographic information such as age, gender, and occupation, as well as details about their workplace, experience, education/training on using the WHO checklist, the adaptation of the checklist to their department, their responsibilities in implementing and using the checklist, the frequency of use in emergency situations, and the impact on patient safety.

Results

The results of the study showed that assistant nurses, despite having the lowest level of education among healthcare professionals, were highly trusted and valued by other members of the surgical team. Most healthcare professionals were unsure who was responsible for using the WHO checklist but believed it was the assistant nurse's responsibility to ensure its implementation. Assistant nurses reported little to no training on using the checklist but noted that it had been adapted to the department's needs. Almost half (48.8%) of assistant nurses believed that the checklist was often used in emergency surgery, and most believed that it improved patient safety.

Conclusions

Improved understanding of the significance of assistant nurses in implementing the WHO Surgical Safety Checklist may enhance adherence to the checklist and potentially improve patient safety, as they were the most valued and trusted healthcare professionals in the surgical team according to the study's findings.

## Introduction

In 2021, the healthcare sector in Sweden was the largest occupational area with over half a million employees, including 300,000 assistant nurses [1]. Although assistant nursing is not a licensed profession, their educational qualifications hold significant importance in the healthcare industry. While there are no national education requirements for assistant nurses, employers generally expect them to have completed an educational program that aligns with the guidelines of the Swedish National Board of Health and Welfare, which recommend at least 1,400 upper secondary school credits in basic care and nursing. This means that students who are interested in pursuing a career in healthcare as an assistant nurse should choose a relevant program during their secondary education, such as healthcare or social care, and accumulate credits by completing courses and passing exams. Each course is assigned a certain number of credits, usually ranging from 50 to 300, depending on the length and complexity of the course [2].

The assistant nurse possesses a wide range of skills and competencies, which enables them to work across various fields. They are integral in providing essential care, support, and services to patients. The areas in which an assistant nurse can work are broad and include elderly care, disability care, psychiatry, and health care, as stated by the Swedish National Agency for Education (2021) [3]. In surgical care, the assistant nurse assumes numerous tasks, such as receiving the patient in the ward with the rest of the team, moving them to the operating table and activating monitoring, inserting catheters, assisting with intubation or spinal anesthesia, arranging the patient for the surgery with the rest of the team, preparing the patient with pre-washing and placing the anesthetic arch on the operating table, assisting the rest of the team with sterile coats, and implementing the World Health Organization (WHO) checklist in collaboration with the team. The Swedish Health Care Act [4] defines health care as the measures taken to medically prevent, investigate, and treat injuries and diseases, with patient safety being the top priority.

While the role of the assistant nurse holds great potential for further research, it remains an intriguing and relatively underexplored field. Only two studies have provided insight into the pathway from education to employment for assistant nurses in Sweden, highlighting the significance of knowledge gained through everyday practice and the importance of collaborating with other healthcare providers. Additionally, these studies have contributed to a better understanding of obstacles and opportunities for learning and development in the healthcare industry, emphasizing the need to participate in both formal and informal learning activities [5,6].

The role of the assistant nurse in implementing the WHO Surgical Safety Checklist is crucial in improving patient outcomes by reducing errors and adverse events, and promoting teamwork and communication during surgery [7]. Studies have shown that the use of this 19-item checklist can significantly reduce postoperative complications, morbidity, and mortality, and is widely used by surgical providers worldwide [8-11]. Adherence to the checklist is positively associated with greater knowledge and experience, highlighting the importance of the assistant nurse's training and competency in using the checklist effectively [11].

However, there are still significant obstacles to appropriate checklist use in practice, such as a lack of knowledge on how to use the list and questioning the value of the list by team members [12]. Several studies published in Sweden have discussed both the positive and negative aspects of using the WHO checklist, with a focus on team dynamics and the varying responsibilities and contributions of healthcare professionals in the checklist implementation [13-15]. Nonetheless, there is a lack of data describing the individual role of the assistant nurse and the use of the WHO checklist in Sweden. Therefore, the aim of this study is to describe the experience of Swedish healthcare professionals in using the WHO Surgical Safety Checklist, with a particular focus on assistant nurses as members of the surgical team.

## Materials and methods

Patients and study design

Between September 2018 and March 2019, a descriptive quantitative study was conducted in two surgical wards at a university hospital located in the western region of Sweden. The survey was conducted among all healthcare professionals employed at the hospital during the data collection period. The inclusion criteria for the study required healthcare professionals who were employed at the hospital, willing to participate in the study, and reported using the WHO checklist at least five times. Healthcare professionals who were hired as extra staff or declined to participate in the study were excluded from the research.

A total of 248 healthcare professionals were eligible to participate, including surgical nurses, nurse anesthetists, assistant nurses, surgeons, obstetric surgeons, and anesthesiologists. Of these, 196 healthcare professionals completed the questionnaire, representing 79% of the eligible participants. Furthermore, 43 assistant nurses, which corresponds to 22% of the eligible participants, were present in the research group. The data for the study were collected from employees working in two different units. Unit one performed surgery for women's diseases, while unit two specialized in surgery for abdominal complaints.

The questionnaire

The authors developed a questionnaire to understand the aim of the study, which was based on literature, their own surgical experience, and the use of the WHO checklist. To validate the questionnaire, one surgical nurse, one nurse anesthetist, one anesthesiologist, and one surgeon were included in the study, and the test-retest statistical method was employed. The healthcare professionals were provided with both written and verbal information about the study and the questionnaire. They were given a two-week period to anonymously answer the questionnaire on their respective wards and received two reminder emails during this time. Completing the questionnaire took approximately five to eight minutes.

The questionnaire (Table 1) included details such as age, gender, occupation, work experience, workplace of the healthcare professionals, completion of education/training on using the WHO checklist at their department, the adaptation of the WHO checklist in their department, clarity on the responsibility of implementing the WHO checklist, the responsibility of using the WHO checklist before surgery, frequency of using the WHO checklist in emergency situations, and the impact of the WHO checklist on enhancing patient safety.

**Table 1:** The six-item questionnaire used in the survey

1. Have you received education or training on the use of the WHO checklist in your department?
Yes
No
2. Has the WHO checklist been adapted to your department?
Yes
No
3. Who is responsible for ensuring that the WHO checklist is implemented?
Surgeon
Anesthesiologist
Nurse anesthetist
Surgical nurse
Assistant nurse
4. Do you know who is responsible for implementing the WHO checklist before surgery?
Yes
No
5. What is your opinion on how frequently the WHO checklist is utilized in emergency situations?
All the time
Often (every second operation)
Seldom (every fourth operation)
Never
6. Does the WHO checklist improve patient safety?
Totally agree
Partly agree
Doubtful
Do not agree at all

Statistical analysis

The data were presented as both absolute (N) and relative (%) numbers. The D'Agostino-Pearson test was employed to analyze the data distribution. For normally distributed data, a comparison between the groups was conducted using Student's t-test, while the chi-square and Fisher's exact tests were used for categorical variables. Statistical significance was considered to be present at p < 0.05. The p-value was adjusted for multiple comparisons using the Bonferroni correction. The statistical analyses were performed using MedCalc Statistical Software for Windows, version 19.0.3. (MedCalc Software, Mariakerke, Belgium).

Ethical considerations

In accordance with Swedish Health Care Act [16], since the study did not involve any information on individual health issues or physical interventions, there was no requirement to seek approval from an ethics committee. The World Medical Association Declaration of Helsinki [17] guidelines were adhered to throughout the study. The identities of the participating healthcare professionals, including their names and personal identity numbers, were not disclosed in any publications or records, ensuring their confidentiality was maintained.

## Results

**Figure 1:**
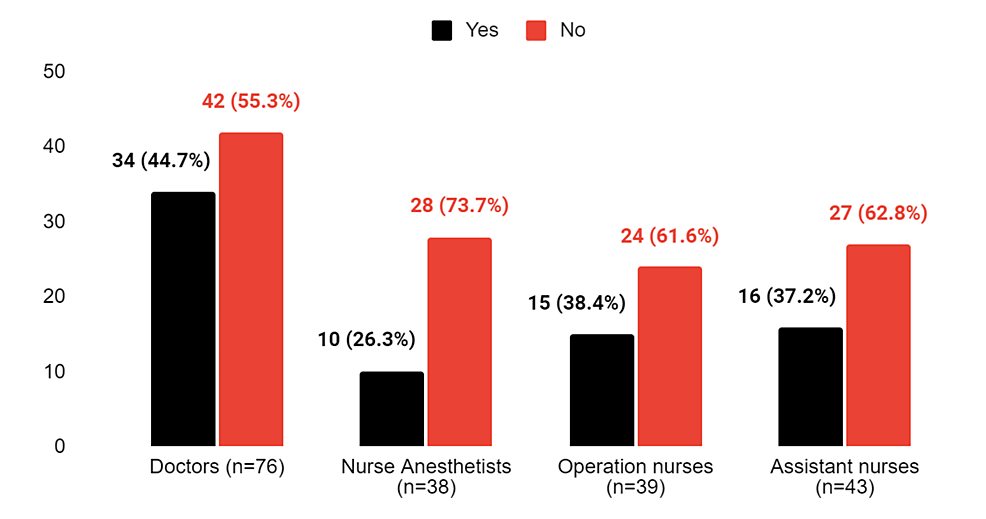
The response to the question "Have you received education or training on the use of the WHO checklist in your department?"

**Figure 2:**
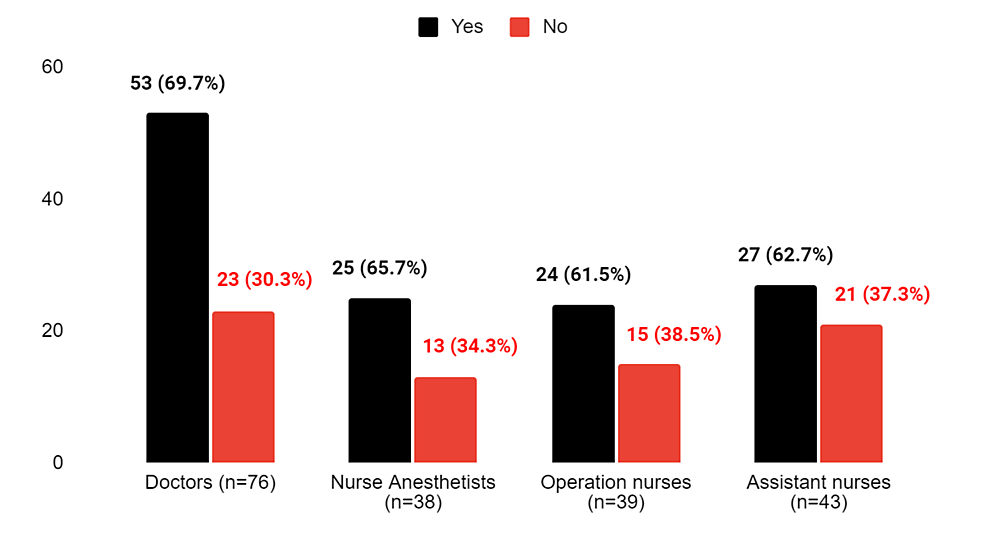
The response to the question "Has the WHO checklist been adapted to your department?"

**Figure 3:**
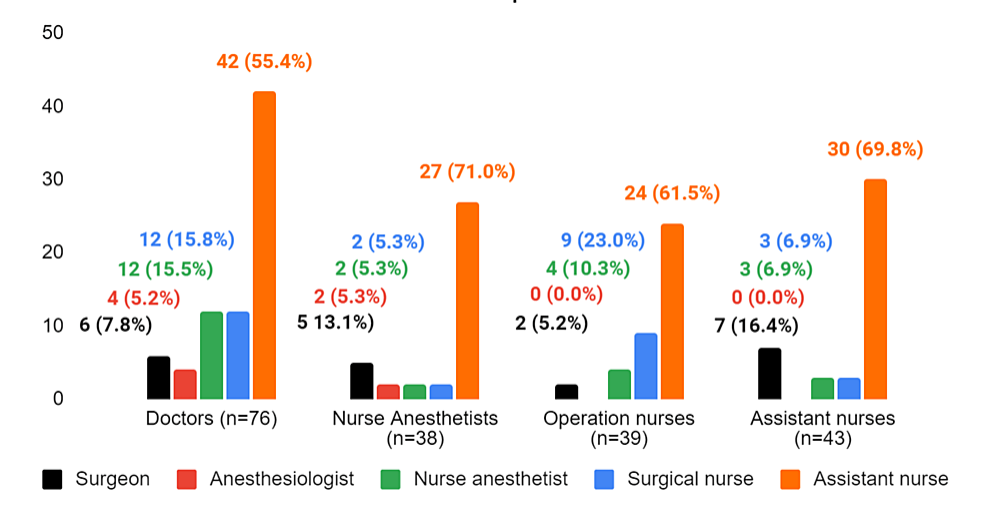
The response to the question "Who is responsible for ensuring that the WHO checklist is implemented?"

**Figure 4:**
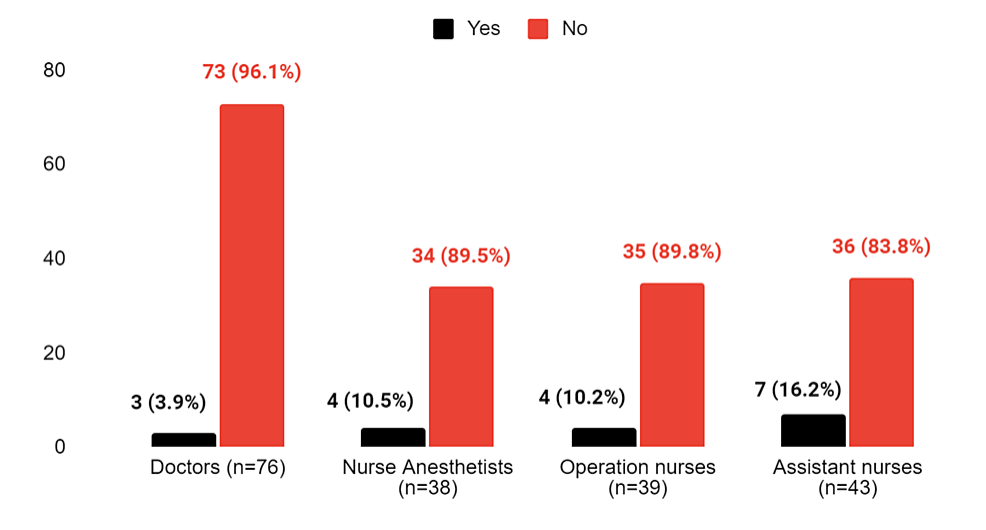
The response to the question "Do you know who is responsible for implementing the WHO checklist before surgery?"

**Figure 5:**
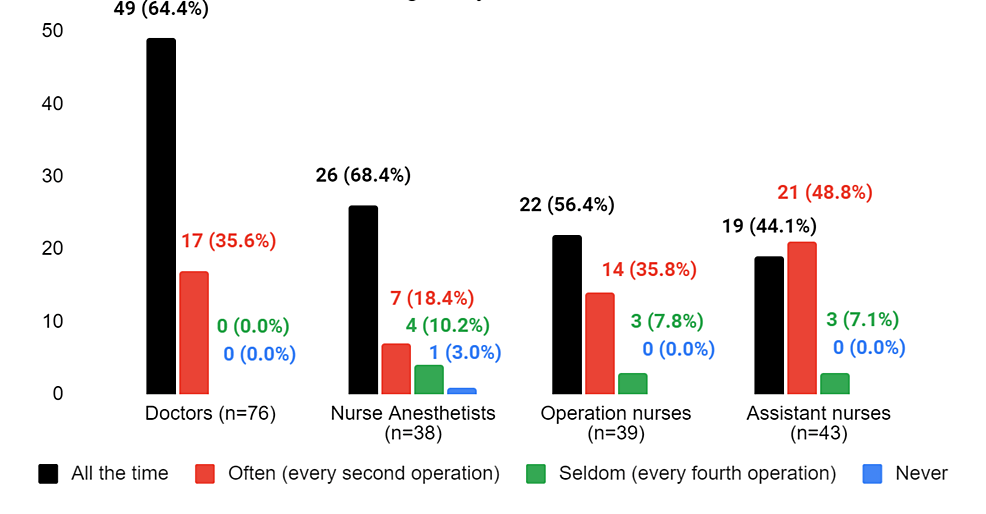
The response to the question "What is your opinion on how frequently the WHO checklist is utilized in emergency situations?"

**Figure 6:**
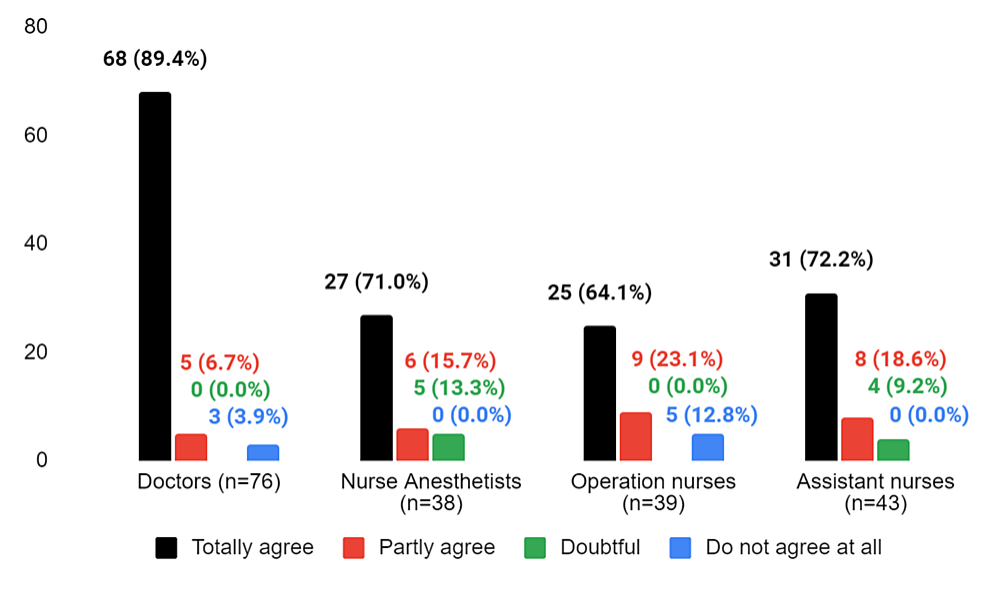
The response to the question "Does the implementation of the WHO checklist result in improved patient safety?"

A total of 196 healthcare professionals completed the questionnaire, with the majority being female (149) and a mean age of 42.4 years. There were also 47 male participants with a mean age of 40.2 years. Most of the participants had over 20 years of work experience (Table 2). Among the respondents, the largest group was assistant nurses, comprising 43 individuals (23%), followed by surgical nurses with 39 participants (20%), and anesthesiologists with 36 individuals (18%). On the other hand, surgeons had the least number of respondents, comprising only 17 individuals (8%). The study results are presented in Figures 1-6, focusing on a comparison between the four different occupations and the experience of using the WHO checklist among assistant nurses.

**Table 2:** Characteristics of participants

Gender	Number (%)
Male	47 (24)
Female	149 (76)
Total	196
Workplace	
Workplace 1	107 (44)
Workplace 2	89 (56)
Total	196
Occupation	
Anesthesiologist	36 (18)
Obstetrics	23 (12)
Surgeon	17 (8)
Nurse anesthetist	38 (19)
Surgical nurse	39 (20)
Assistant nurse	43 (23)
Total	196
Age (years)	
21-30	31 (16)
31-40	43 (23)
41-50	55 (28)
51-60	49 (25)
≥60	21 (11)
Total	196
Experience (years)	
≤5	54 (28)
6-10	30 (15)
11-15	22 (12)
16-20	23 (12)
≥20	67 (33)
Total	196

Most participants reported not receiving any training on the use of the checklist, with nurse anesthetists having the highest percentage (73.7%) in this regard. Despite this, doctors provided the most positive answers at 44.7%, while 61.6% of surgical nurses responded negatively. Assistant nurses had the second-lowest positive response rate at 37.2% (Figure 1). Assistant nurses differ significantly from the other three groups of healthcare professionals (p = 0.011) and individually from doctors and nurse anesthetists in terms of completion of education/training on using the WHO checklist in their department (p = 0.032 and p = 0.00028, respectively).

Regarding the adaptation of the WHO checklist to their departments, doctors had the highest positive response rate at 69.7%, while nurse anesthetists and other occupations had negative responses to the second question. Surgical (operation) nurses had the highest positive response rate among other staff members, with 61.5% stating that the checklist was well adapted to their department. In addition, 62.7% of assistant nurses also reported that the checklist had been adapted to their department (Figure 2). Assistant nurses differ significantly from the other three groups (p = 0.009) and individually from doctors in terms of adaptation of the WHO checklist to their department (p = 0.008). However, there is no significant difference between assistant nurses and nurse anesthetists or operation nurses.

Regardless of their profession, the majority of participants felt that assistant nurses were responsible for ensuring the use of the checklist. Nurse anesthetists (71.0%) and doctors (55.4%) were the groups most likely to place this responsibility on assistant nurses. All surgical nurses provided similar responses to other staff members (61.5%), stating that assistant nurses were responsible for ensuring the use of the checklist. Two-thirds of assistant nurses felt that their profession was responsible for ensuring the use of the checklist (69.8%) (Figure 3). There is no significant association between assistant nurses and other occupations in terms of their responses to this question.

However, many participants felt that it was not clear who was responsible for ensuring the use of the WHO checklist before operating on a patient. Only 16.2% of assistant nurses stated that they knew who was responsible for the use of the checklist before an operation, while 96.1% of all doctors reported that they did not know who was responsible (Figure 4). However, there is no significant difference between the groups in terms of their responses to this question (p = 0.107).

A majority of nurse anesthetists (68.4%) reported that the checklist was used in emergencies, as did most of the other participants. Among assistant nurses, 44.1% stated that the checklist is always used in emergencies. Not a single assistant nurse stated that the checklist is never used during emergency situations (Figure 5). There is a significant difference between assistant nurses and doctors or nurse anesthetists in their responses to the statement, with doctors and nurse anesthetists being less likely to think that the WHO checklist is used in emergency situations (p = 0.0112 and p = 0.0252, respectively). There are no other significant differences between the groups.

Most doctors (89.4%) believed that the checklist contributed to improved safety, as did other participants regardless of their current profession. Among nurse anesthetists, 13.3% were unsure about the list, while surgical nurses stated that it would improve patient safety. However, four (9.2%) assistant nurses were hesitant about whether the checklist improves patient safety (Figure 6).

Assistant nurses differ significantly from doctors in their responses, with assistant nurses more likely to agree with the statement (p = 0.0268). However, there is no significant difference between assistant nurses and nurse anesthetists or operation nurses in their responses to this question/statement (p = 0.0536 and p = 0.2856, respectively).

## Discussion

The purpose of this study was to investigate the experience of Swedish healthcare professionals in implementing the WHO Surgical Safety Checklist, specifically emphasizing the role of assistant nurses as members of the surgical team. WHO Surgical Safety Checklist is a tool developed by the WHO to improve patient safety during surgery and reduce the incidence of surgical errors and complications. It is divided into three parts: before anesthesia is administered, before the incision is made, and before the patient leaves the operating room. The checklist includes items such as verifying the patient's identity and the site of the surgery, confirming that all necessary equipment is available and in working order, and ensuring that the surgical team has reviewed critical information such as the patient's allergies and medical history [7].

The results indicate that, despite having the least amount of training among all surgical staff categories, assistant nurses were the most appreciated and trusted by other healthcare professionals in the surgical team. Although the majority of participants did not know who was responsible for using the WHO checklist before surgery, most respondents, including assistant nurses themselves, indicated that it was the assistant nurse's responsibility to ensure that the WHO checklist was implemented. The study's findings demonstrate that assistant nurses are highly valued by their colleagues, which is undoubtedly positive. However, claiming that they were solely responsible for implementing the checklist without knowing the actual personnel in charge is not a point of pride.

In our opinion, the issues with the implementation of the WHO Surgical Safety Checklist are related to organizational shortcomings, which may be influenced to some degree by the hierarchy in surgical departments. To ensure that patients and staff members using the checklist feel comfortable with its use, all departments should regularly review the list, identify who uses it the most, and ensure that all questions are read aloud for each patient. Studies have shown that the successful implementation of the checklist is dependent on organizational and socio-cultural factors. Our data analysis suggests that regular organizational changes are necessary, including specific schedules for implementing the checklist and allowing enough time for thorough reviews of all items. Sociocultural factors, such as professional relationships and cultural habits, also play a role. A safety culture must be established in the organization, with coordinated efforts from everyone involved in reducing risks in surgical care. Full support for the checklist from all managers in the surgical process is essential, and regular follow-ups are crucial. However, achieving this goal remains only an ambition in the current context, where many health managers are undertrained, and there is a persistent staff shortage at all levels, from cleaners to top-level medical professionals [9,18,19]. Regarding the hierarchy, our previous studies on the same subject and with the same staff have shown that the acceptance of the list varied depending on coworkers [13].

Other studies have found that the frequency and quality of checklist implementation decrease with the age of staff, with older surgeons and anesthesiologists being the least engaged in using the checklist [20]. In 2011, Thomassen et al. [21] emphasized in their research that some experienced doctors were skeptical about using the checklist, remarking that after many decades of performing their duties without it, they now had to "play" with a checklist. The study also noted that the support and motivation of the chief physician were crucial for checklist use and that a negative chief physician could pose challenges to adequate implementation. Like the majority of participants in the present study, assistant nurses reported a lack of formal training in using the checklist, but they appreciated the adaptation of the checklist to their department. In another Swedish study, only 43% of participants had received training in using the WHO checklist. Other similar studies have explained that inadequate implementation may be due to insufficient knowledge among participants about how to use the checklist, highlighting the need for well-structured training [22-24].

Although the majority of respondents reported that the WHO checklist was always used for emergency patients, 48.8% of assistant nurses believed that it was used more frequently. This suggests that assistant nurses may have the most control over the checklist process. Treating both elective and acute patients can lead to different types of errors, and stress associated with emergency operations can be mitigated by using the WHO checklist. In a 2010 interview study by Nilsson et al. [20], all participants stressed the importance of using the WHO checklist for both planned and emergency cases. In another study from the United States, surgeons reported more system errors, such as communication breakdowns and increased workload, in emergency surgeries compared to planned surgeries [25]. This finding may support our results showing that almost half of the assistant nurses believe the WHO checklist is not used as regularly as it should be for emergency operations, potentially due to staffing and organizational shortages. All authors of the present study reflected on the situation a few decades ago, specifically referencing a well-known nursing theorist [26], and highlighted the importance of dialogue occurring both peri- and postoperatively.

The perioperative dialogue involves nurse anesthetists, operating room nurses, doctors, and patients engaging in a series of three encounters. Its objective is to foster a sense of community and provide space for communication through dialogue [27]. Prior to surgery, the preoperative dialogue takes place between the nurse and the patient. During this encounter, the patient has an opportunity to share their story, expectations, and thoughts about the surgery and anesthesia, while also getting to know the nurse. The nurse's role is to actively listen, address the patient's questions and needs, and explain any relevant details. Through this exchange, the nurse and patient can plan the intraoperative period together. The intraoperative dialogue commences when the patient and the same nurse meet in the operating room. The nurse then implements the plan and explains the procedure to the patient. Finally, during the postoperative dialogue, the same nurse checks on the patient, and together, they evaluate the care provided by the nurse by discussing the patient's experience. Throughout the perioperative dialogue, the nurse's caring attitude and responsibility toward the patient are highlighted.

Professional nurses have the responsibility of creating a nurturing relationship with their patients, instilling confidence in them, and fulfilling their ethical obligations [28]. Although these principles have been established for over three decades, they may only represent an ideal in today's Swedish healthcare system. The majority of assistant nurses, like other healthcare professionals, believe that the WHO checklist improves patient safety. A study with 331 participants found that 93% of them agreed that the checklist was beneficial, while only 1% did not think it contributed at all. Interestingly, the authors noted that there were no differences in opinions between various healthcare professionals [21]. While the WHO checklist cannot replace the expertise and skills of healthcare professionals, they are the ones who use it on a daily basis. It is uncertain where the healthcare system is heading when the critical and responsible role in the operational team is carried out by the lowest-ranking personnel, who are also decreasing in number and are at risk of becoming extinct.

The present study had some limitations, such as the low response rate, which could be attributed to the fact that it was the first of its kind conducted in our hospital. Despite providing a detailed outline of the survey process, employees expressed concerns about anonymity. Lastly, the study only examined compliance with the use of checklists and did not evaluate the quality of their usage.

## Conclusions

The findings of the current study suggest that, despite being the least trained healthcare professionals in the operating room and surgical process, assistant nurses were the most valued and trusted by all other healthcare professionals in the surgical team. Although most respondents were unsure about who was responsible for implementing the WHO checklist before surgery, including assistant nurses themselves, they believed that it was the assistant nurse's responsibility. Despite feeling that they lacked training in using the checklist in their department, assistant nurses were satisfied with how the list had been adapted to meet the specific needs of their department. While most participants believed that the checklist was only used for emergency patients, nearly half of the assistant nurses believed that it was used more frequently. Nevertheless, the majority of assistant nurses believed that the WHO checklist contributed to improved patient safety.
